# Exploring changes in the perception of e-professionalism among medical and dental students: a quantitative cross-sectional study

**DOI:** 10.3325/cmj.2024.65.43

**Published:** 2024-02

**Authors:** Danko Relić, Marko Marelić, Joško Viskić, Lovela Machala Poplašen, Marjeta Majer, Kristijan Sedak, Tea Vukušić Rukavina

**Affiliations:** 1 Andrija Štampar School of Public Health, University of Zagreb School of Medicine, Zagreb, Croatia; 2 University of Zagreb School of Dental Medicine, Zagreb, Croatia; 3 Catholic University of Croatia, Zagreb, Croatia; 4 Biomedical Research Center Šalata, School of Medicine, University of Zagreb, Zagreb, Croatia

## Abstract

**Aim:**

To compare e-professionalism perceptions between medical and dental students, focusing on their awareness and understanding of guidelines for developing e-professionalism.

**Methods:**

A cross-sectional quantitative study was conducted at the University of Zagreb School of Medicine (UZSM) and School of Dental Medicine (UZSDM) in 2022/2023. Data were gathered through a questionnaire designed specifically for the survey.

**Results:**

Of the 646 questionnaires collected, 626 were analyzed, with a response rate of 33.95% for UZSM and 37.83% for UZSDM. Most respondents (71.4%) were female, with a median age of 21. Medical students significantly more frequently considered it unprofessional to publish posts containing photos of patients/clients (96.5% vs 75.1%), endorsements of health products without conflict-of-interest disclosures (60.6% vs 33.0%), and posts describing patient interactions without revealing identifying information (51.7% vs 27.4%). In contrast, dental medicine students more frequently considered it unprofessional to publish posts with swearing or foul language (81.2% vs 67.4%), critical comments about lecturers (68.0% vs 46.9%), and criticisms of course material or the institution (52.3% vs 36.4%). Only 23.2% of students were aware of e-professionalism guidelines, with 37.9% of those familiar with their content.

**Conclusion:**

While medical and dental students recognize the importance of e-professionalism, their perceptions have substantial differences. The need for promoting existing guidelines and integrating e-professionalism into curricula is evident. Continuous monitoring and research in this domain are essential to ensure future health care professionals maintain high standards of online professionalism.

The digital age has brought about a paradigm shift in how individuals communicate, learn, and share information. This transformation is particularly evident in higher education, where students increasingly leverage digital platforms, mainly social media (SM), for academic and professional purposes (1). The biomedical field, which encompasses disciplines like medicine and dentistry, is no exception to this trend.

With their widespread influence and interactive nature, SM platforms grant students distinct chances for group learning, accessing expert insights, and promoting entrepreneurial activities (2). Yet, weaving these platforms into academic and professional settings comes with challenges. While SM can enhance personal and professional identities, they blur the lines between the two, which raises concerns about e-professionalism (3).

E-professionalism has been defined as encompassing “attitudes and behaviors, some of which may transpire in private settings. These behaviors reflect traditional professionalism paradigms and are exhibited through digital media” (4).

Both medicine and dental medicine uphold foundational ethical principles, such as prioritizing the patient’s best interests, maintaining confidentiality, and respecting autonomy (5-7). While the foundation may be the same, the distinctions are emerging rapidly, with dentistry being a more visual field in education and patient interactions and growing into a more private practice-oriented field (5,6).

Since the beginning of e-professionalism research, the scientific community has struggled to define, quantify, and evaluate e-professionalism (3,8-13). Marelić et al introduced the systematically designed SMePROF-S scale to assess medical and dental students’ attitudes toward e-professionalism (13). Their significant contribution aligns with an expanding international and national discourse, particularly in Croatia, where scholarly inquiries into the perceptions and engagement of medical and dental students with e-professionalism are becoming more prevalent (11,13-15).

We have previously published research results about SM use, habits, and attitudes toward e-professionalism using the questionnaire “Exploring the Impact of Social Networks on the Professional Behavior of Healthcare Professionals” on students of both schools (16). Another factor has also influenced the decision to revisit this student population. Our previous research was conducted in 2018/2019, and the results were published in 2021 (16). Many changes in the functioning and usage of SM have occurred since, leading to potential new insights to be discovered in this research (17). Therefore, this study aims to compare e-professionalism perceptions between medical and dental students, focusing on students’ awareness and understanding of the guidelines designed to encourage the development of e-professionalism during their studies.

## Participants and methods

This quantitative cross-sectional study was conducted at the University of Zagreb School of Medicine (UZSM) and School of Dental Medicine (UZSDM) in 2022/2023. Data were gathered through a survey instrument referred to as “Exploring the Impact of Social Networks on the Professional Behavior of Healthcare Professionals” (16).

The questionnaire was accessible on the official websites of the medical and dental school project from October 2022 to July 2023, spanning the academic year 2022/23 (18). Students in the first, second, fifth, and sixth study year at UZSM and all study years at UZSDM were notified about the opportunity to participate in the study during regular classes. Participation was voluntary, with no incentives provided for completing the survey. To ensure confidentiality, no personal identification information was gathered. Google Forms did not retain data by default if the questionnaire was left incomplete. Duplicate entries were removed, and respondents who indicated non-use of SM had their negative responses recorded. They were then redirected to the end of the questionnaire, and their responses were excluded from the analysis.

The instrument used to measure the perception of e-professionalism was initially developed by White et al and included a total of 19 items (19). Subsequently, Marelić et al translated and modified this instrument for use in the Croatian language (13). Viskić et al introduced the ePACI index, derived from responses to the White et al instrument (20). The theoretical spectrum of this index extends from -1 to +1. Negative scores suggest a deviation toward a “liberal” orientation, where unprofessional conduct is viewed as acceptable. Conversely, positive scores denote a deviation toward a “conservative” stance, where acceptable behavior is seen as unprofessional. Values near the midpoint of the range (zero) reflect a perception that aligns with the norm (20). Importantly, the labels “liberal” and “conservative” do not inherently convey positive or negative connotations. Any difference in index values from zero, whether leaning toward the liberal or conservative side, signifies a deviation from the accurate understanding of e-professionalism (20). Viskić et al comprehensively explain the mathematical and statistical processes of creating this index (20). The study was approved by the Ethics Committees of the UZSM and the UZDSM.

### Statistical analysis

The normality of the index distribution was tested with the Kolmogorov-Smirnov test. The index deviation from the norm was tested with a one-sample *t* test. Descriptive statistics were applied to analyze demographic data. Differences between the groups were assessed with a χ^2^ test and a Mann-Whitney U test. For the 2 × 2 contingency χ^2^ test, Yates's correction for continuity was utilized. The Bonferroni correction was used in the case of multiple comparisons within the same instrument. Statistical analysis was performed with SPSS Statistics, version 26 (IBM Corp., Armonk, NY, USA).

## Results

A total of 646 questionnaires were collected. Three respondents did not give informed consent, and since no school/study-year data was collected from them, they were not included in the response rates. The response rates were 33.95% for UZSM and 37.83% for UZSDM. Furthermore, 20 respondents were excluded because they stated that they did not use any SM. A total of 626 respondents were included in the study, 429 UZSM and 197 UZSDM students. The sample was predominantly female (71.4%), with a median age of 21. The distribution of the participants by sex and study years is shown in Table 1.

**Table 1:** The distribution of the participants’ sex and study years (N = 626)

	No. (%) of students		
	all	medical	dental
**Sex**			
male	179 (28.6)	143 (33.3)	36 (18.3)
female	447 (71.4)	286 (66.7)	161 (81.7)
total	626 (100)	429 (100)	197 (100)
**Study year**			
first	139 (22.2)	95 (22.1)	44 (22.3)
second	163 (26.0)	101 (23.5)	62 (31.5)
third	44 (7.0)	0	44 (22.3)
fourth	16 (2.6)	0	16 (8.1)
fifth	120 (19.2)	106 (24.7)	14 (7.1)
sixth	144 (23.0)	127 (29.6)	17 (8.6)
**Total**	626 (100)	429 (100)	197 (100)

The majority of respondents believed that posts featuring the following content were considered unprofessional: information about a patient/client (97.4%; 97.5% dental vs 97.4% medical), petty criminal activity (92.7%; 94.9% dental vs 91.6% medical), and illicit drug consumption (90.6%; 92.4% dental vs 89.7% medical).

Compared with dental medicine students, medical students significantly more frequently considered certain types of content as unprofessional: photos of a patient/client (96.5% vs 75.1%; *P* < 0.001), endorsements of pharmaceutical or health products without a conflict-of-interest disclosure (60.6% vs 33.0%; *P* < 0.001), and descriptions of interactions with a patient/client without revealing any identifying information (51.7% vs 27.4%; *P* < 0.001). On the other hand, dental medicine students more frequently regarded the following types of posts as unprofessional: the use of swearing or foul language (81.2% vs 67.4%; *P* < 0.001), critical comments about a lecturer or preceptor (68.0% vs 46.9%; *P* < 0.001), and critical comments about course material, program, faculty, or the university (52.3% vs 36.4%; *P* < 0.001) (Table 2).

**Table 2:** Medical and dental students' responses regarding what posts they consider unprofessional on social media (N = 626)

Posts disclosing	No. (%) of students			χ^2‡^; df; P
	all	medical	dental	
Information about a patient/client	610 (97.4)	418 (97.4)	192 (97.5)	0.000; 1; 1.000
Petty criminal activity	580 (92.7)	393 (91.6)	187 (94.9)	1.720; 1; 0.190
Illicit drug consumption	567 (90.6)	385 (89.7)	182 (92.4)	0.816; 1; 0.366
Involving overt sexual content	566 (90.4)	378 (88.1)	188 (95.4)	7.523; 1; 0.006
Photos of a patient/client	562 (89.8)	414 (96.5)	148 (75.1)	64.905; 1; <0.001†
Attitudes of superiority or condescending behavior (assumed because of professional status)	490 (78.3)	340 (79.3)	150 (76.1)	0.597; 1; 0.440
Swearing or foul language	449 (71.7)	289 (67.4)	160 (81.2)	12.774; 1; 0.001*
Obscene gestures in photos (the middle finger, etc)	439 (70.1)	288 (67.1)	151 (76.6)	5.391; 1; 0.020
Pictures of an individual clearly behaving drunkenly	425 (67.9)	293 (68.3)	132 (67.0)	0.053; 1; 0.818
Partial nudity	399 (63.7)	259 (60.4)	140 (71.1)	6.224; 1; 0.013
Substantial alcohol consumption at a party	387 (61.8)	264 (61.5)	123 (62.4)	0.016; 1;0.900
Critical comments about a lecturer or preceptor	335 (53.5)	201 (46.9)	134 (68.0)	23.472; 1; 0.001*
Endorsements of a pharmaceutical or health product without a conflict-of-interest disclosure	325 (51.9)	260 (60.6)	65 (33.0)	40.132; 1; <0.001†
Membership in online groups dealing with controversial issues	302 (48.2)	191 (44.5)	111 (56.3)	7.092; 1; 0.008
An interaction with a patient/client, while not revealing any identifying information	276 (44.1)	222 (51.7)	54 (27.4)	31.459; 1; <0.001†
Critical comments on course material, your program, faculty, or the university	259 (41.4)	156 (36.4)	103 (52.3)	13.459; 1; <0.001†
Making opinionated comments about controversial issues	250 (39.9)	158 (36.8)	92 (46.7)	5.080; 1; 0.024
A picture of an individual having one alcoholic beverage	129 (20.6)	90 (21.0)	39 (19.8)	0.054; 1; 0.816
Displaying your current relationship status	59 (9.4)	38 (8.9)	21 (10.7)	0.324; 1; 0.569

*P < 0.05 after Bonferroni correction for 17 comparisons.

†*P* < 0.01 after Bonferroni correction for 17 comparisons.

‡Yates's correction for continuity.

A small proportion of students, 9.4% (8.9% medical vs 10.7% dental), considered it unprofessional to display current relationship status, to post a picture of an individual having one alcoholic beverage (20.6%; 21.0% medical vs 19.8% dental), and to post opinionated comments about controversial issues (39.9%; 36.8% medical vs 46.7% dental) (Table 2).

Among the surveyed students, 23.2% (n = 145) were aware of the guidelines for developing e-professionalism, with a slightly higher awareness rate among medical students (24.5% medical vs 20.3% dental). Of those aware of the guidelines, 37.9% (n = 55) were aware of the content, and of these, 47.3% (n = 26) stated that the guidelines changed how they used SM (Table 3).

**Table 3:** Awareness of the existence of guidelines, their content, and self-assessment of their impact

No. (%) of students				χ^2^*; df; P
all		medical	dental	
Awareness of the existence of guidelines (n = 626)				
yes	145 (23.2)	105 (24.5)	40 (20.3)	1.096; 1; 0.295
no	481 (76.8)	324 (75.5)	157 (79.7)	
Familiarity with the content of the guidelines (n = 145)				
yes	55 (37.9)	44 (41.9)	11 (27.5)	1.978; 1; 0.160
no	90 (62.1)	61 (58.1)	29 (72.5)	
Guidelines had an impact on changing their behavior on social media (n = 55)				0.770; 1; 0.380
yes	26 (47.3)	19 (43.2)	7 (63.6)	
no	29 (52.7)	25 (56.8)	4 (36.4)	

*Yates's correction for continuity.

There was no significant difference between medical and dental students in their awareness of the existence of guidelines (χ^2^ = 1.096; df = 1; *P* = 0.295), familiarity with the content of guidelines (χ^2^ = 1.978; df = 1; *P* = 0.160), or in their self-assessed impact of the guidelines on their behavior on SM (χ^2^ = 0.770; df = 1; *P* = 0.380). Additionally, there was no significant difference in familiarity with the guidelines based on the year of study (χ^2^ = 8.206; df = 5; *P* = 0.145).

The ePACI index, employed following the methodology outlined in Viskić et al (20), was used to assess e-professional behavior. The index's scale extended from -1 to 1, exhibiting an average of 0.17 (with a standard deviation of 0.446). Our sample's responses significantly diverged from the theoretically “neutral” answer, with a notable shift toward positive “conservative” values (*t* = 28.022; df = 625; *P* < 0.001). The ePACI index was not normally distributed (D = 0.079; df = 626; *P* < 0.001).

Although both medical and dental students showed a significant positive or “conservative” deviation, dental students (mean rank = 334.58) had a more significant deviation, indicating a more conservative perception of e-professionalism (mean rank = 303.82; U = 38103.5; *P* = 0.047).

There was no significant difference in the ePACI index results between students who were aware of the existence of guidelines and those who were not (U = 33530; *P* = 0.481). Furthermore, there was no significant difference in the ePACI index between students who were familiar with the content of the guidelines and those who were not (U = 2308.5; *P* = 0.496). Similarly, there was no difference between those who believed the guidelines had influenced their behavior and those who did not (U = 312; *P* = 0.270).

Students in the 2022/23 wave exhibited a significantly more cautious or “conservative” perception of e-professionalism (mean rank = 684.78) compared with students in the 2018/19 wave (mean rank = 642.52; U = 204529.5; *P* = 0.044) (Table 4).

**Table 4:** Differences in the ePACI index between 2018/19 and 2022/23 wave

Wave	n	Mean (standard deviation)	Mean rank	U; P
2018/19	698	0.12 (0.463)	642.52	204529.5; 0.044
2022/23	626	0.17 (0.446)	684.78	

Since some of the students who were in the 2018/19 wave of the study as second-year students were present in the 2022/23 sample as sixth-year students, to test for bias, the difference was tested in a sample without second-year students from the 2018/19 wave and sixth-year students from the 2022/23 sample (N = 945). Even without these students, the direction and significance of the difference in the ePACI index remained unchanged (U = 100479.5; *P* = 0.008), which indicates that multicollinearity was not an issue.

## Discussion

In our research, SM were widely used throughout the entire sample. Also, medical and dental medicine students showed comparable SM patterns and had similar views on e-professionalism. This aligns with expectations when considering the legal and ethical context of these studies, and it further expands upon the findings from research by Viskić et al (16).

While previous research has touched upon the general use of SM and its implications for professionalism (21-23), our study examined more closely the specific variations of how these students perceived and engaged with e-professionalism. One of the prominent findings is the comparable SM patterns and views on e-professionalism between medical and dental students, which suggests a shared understanding and approach to e-professionalism, likely influenced by the legal and ethical contexts of their respective fields. This continues our research groups’ previous results and underlines the need for collaboration in developing guidelines and educational efforts (16). Such insights are pivotal, especially when considering the increasing integration of digital platforms in health care education and practice. However, this research also highlights the differences in perceptions between these two student groups.

These differences were visible in two distinct areas. Medical students showed an increased sensitivity toward patient-related content. Compared with their dental counterparts, they significantly more often considered photos of patients/clients, endorsements of health products without conflict-of-interest disclosures, and posts describing patient interactions as unprofessional. On the other hand, dental students showed a broader concern for professionalism in terms of language and institutional respect. They more frequently considered posts with swearing or foul language, critical comments about lecturers, and criticisms of course material or the institution as unprofessional. This might indicate an increased awareness among dental students about the importance of maintaining respectful online behavior, especially concerning their educational environment. Interestingly, when it came to personal content, such as displaying current relationship status or sharing a picture with an alcoholic beverage, the differences were minimal. However, dental students were more likely than medical students to view opinionated comments on controversial issues as unprofessional. This could reflect varying professional cultures, educational emphases, or the unique challenges each group faces in interacting with patients and the public.

Chretien et al highlighted the popularity of Web 2.0 applications, such as social networking sites, and the associated risks of broadcasting unprofessional content online (8). Their study revealed that 60% of the surveyed US medical schools had incidents of students posting unprofessional content online. Notably, 13% of these schools reported patient confidentiality violations. Other commonly reported unprofessional behaviors included profanity, discriminatory language, depiction of intoxication, and sexually suggestive material (8). In comparison, our research provides a more detailed view of the specific types of content on SM that medical and dental students perceive as unprofessional. Our results resonate with the study by Chretien et al (8), with a substantial number of students recognizing the pitfalls associated with inappropriate sharing of patient information. This might be attributed to growing awareness and educational initiatives, a stance also supported by Kind et al, who proposed guidelines for medical educators leveraging SM (24).

Viskić et al investigated the perceptions and attitudes of medical and dental students regarding e-professionalism (16). The results revealed that merely 22.3% of the participants believed it was consistently achievable to uphold professionalism in online activities. Notably, a substantial 64.2% reported that their online activities had no impact on their professional behavior. This relaxed attitude toward online professionalism was more pronounced among dental students, with 68.3% endorsing such views compared with 60.8% of their medical counterparts. Moreover, dental students were more inclined toward future patient communication via SM platforms (16). Our study also observed differences in the perceptions of online professionalism between medical and dental students. While both groups recognized the potential pitfalls of unprofessional content, their thresholds and areas of concern differed. This divergence might be attributed to differences in their motivation toward career choice, educational process, and patient interactions (5-7).

Our results gain further relevance when viewed in the context of the #MedBikini movement (25-27). This movement emerged as a response to a study that labeled photos of medical professionals in swimwear as “unprofessional” (25-27). Medical professionals worldwide posted pictures in swimwear on SM to challenge and redefine outdated notions of professionalism. The movement emphasized that personal expressions or attire outside work do not determine competence or professionalism. The #MedBikini movement may have played a pivotal role in fostering a positive shift in the digital conduct of HCPs. Vukušić Rukavina et al further emphasizes context’s roles (12). This study further pointed out a strong comprehension of e-professionalism among both medical and dental students and faculty, with limited instances of unprofessional content observed on public Facebook profiles. These findings signify a progressive and evolving perspective on professionalism within the realm of SM (12).

The alignment of our findings with the #MedBikini movement (25,27) and the mentioned studies or publications underscores a broader trend (12,25-27). While there is consensus on the unprofessional nature of posts violating patient confidentiality, there is a pushback against traditional and potentially restrictive views of professionalism, especially those that may be influenced by gender biases or personal judgments.

Greysen et al highlighted online professionalism as an extension of real-world professionalism (21). This is further emphasized by the disparities in the perceived unprofessionalism of posts containing swearing or critical comments about academia in our study. Students' familiarity with professionalism guidelines in the digital environment needs to improve, as observed in our study, a sentiment shared by Osman et al in their exploration of the generational gap and online professionalism (28).

The difference in the ePACI index between medical and dental students was significant. Contrary to the 2019 results, dental students in the current study showed a more conservative stance (16,20). This shift should be contextualized within the framework of creating guidelines and their integration into the curriculum. While the medical students maintained their previously observed cautious and conservative approach, there was a marked transformation in the attitudes of dental students compared with findings from 2019 (16,20).

The perception of e-professionalism among medical and dental students has recently seen a positive shift. This transformation is partly due to the proactive measures taken by educational institutions, particularly integrating e-professionalism topics into their curricula. Such initiatives have given students insights into the advantages and challenges of maintaining a professional online presence.

SM present unique challenges when it comes to upholding professional values, behaviors, and identities, primarily due to the online disinhibition effect and the far-reaching and enduring digital footprints they create. The blurred boundaries between personal and professional identities in the digital space call for guidelines for appropriate online conduct (29). While creating guidelines for developing e-professionalism among medical and dental students has been a significant step forward, more than their mere existence is needed; the ongoing emphasis on their importance is crucial. The awareness of guidelines remains relatively low, yet those familiar with them often acknowledge their influence on online behavior. These guidelines offer a roadmap for e-professionalism, but it is imperative to underscore their value consistently. White et al further emphasized the need for comprehensive e-professionalism education and awareness to instill these values among students (19). They should be well-informed about these guidelines and receive education on their reasoning, which would ensure a deeper understanding and adherence.

The traditional boundaries separating personal and professional spheres are evident in the physical world. However, in the online realm, the absence of such clear boundaries leads to a merging of lines between entirely different contexts. Diverse approaches may be implemented to alleviate this circumstance, including self-disclosures, self-censorship, tailored digital adjustments for specific audiences, and privacy settings. However, instances may still arise where one's online presence diverges from one's values, competencies, behaviors, and identities.

The primary limitation of this study stems from volunteer bias and the restricted sample selection, as not all medical and dental schools in Croatia were encompassed. Consequently, the findings may lack generalizability to all medical and dental schools across Croatia. The second limitation is that students who were part of the 2018/19 wave sample as second-year students were included in the 2022/23 sample as sixth-year students. Due to EU General Data Protection Regulation, it was impossible to collect identifying information that would allow us to link responses of the same individuals between the two waves. To mitigate bias, a test for differences in the ePACI index was rerun on a sample excluding those years that could have contained the same participants (second-year students of the first wave and sixth-year students of the second wave). Since there was no change in the direction or statistical significance in the repeated test, we believe multicollinearity was not a concern and did not introduce errors in the conclusions.

In conclusion, the digital era presents opportunities and challenges for delineating personal and professional identities, especially for medical and dental students. Our findings emphasize the urgent need for a more robust promotion of existing e-professionalism guidelines (30). The curricula of educational institutions for HCPs require timely revisions to encompass comprehensive e-professionalism training. This ensures that students are prepared to navigate the intricacies of online interactions. Given the swift evolution of digital communication and the ever-changing landscape of SM, there is a need for ongoing monitoring and research in this domain. By maintaining a consistent focus on evaluation and adaptation, we can safeguard the commitment of future HCPs to uphold the highest standards of professionalism in all spheres of their practice.
